# Moral and Physical Massage

**Published:** 1887-04-23

**Authors:** Stretch Dowse


### Moral and Physical Massage.

By Dr. Stretch Dowse.

A Lecture delivered at the Midwives' Institute and Trained Nurses' Club. (*Continued from p. 38.*)

### Part III.—Forms of Massage.

My demonstrations will now be carried on by the aid of the human body, and my first demonstration will be *effleurage.*

I now have my right hand in the position for effleurage. I place my fingers and my thumb, which you see are fully extended upon this man's chest, and I draw them slowly and lightly from above downwards. I now make a return movement quickly with the palm of my hand, and I again make my first movement, as you see, with the tips of my fingers and with the outer edge of my thumb. These manipulations, constituting effleurage, are made with the tips of the fingers and the palm of the hand; this form of stroking movement with the fingers may be done softly or lightly, or with considerable energy. You will find that effleurage performed as lightly as possible after petrissage, for some few minutes, will have a decidedly soothing influence, and a vast deal of vital energy can unquestionably be thrown into the body of the patient in this way. Such forms of massage, when gently and continuously carried over the forehead, the closed eyes, and the cheeks, carry the patient from a state of mere repose to hypnotism, and he not unfrequently passes from this state into one of tranquil sleep. You will, if you please, perceive that I never absolutely remove my hands from the patient's body, and in every form of massage you must, as far as possible, pay attention to this. Your centripetal movements, or those which are directed towards the centre of the body, are those in which the greatest amount of energy is and should be exerted, because, as I before told you, they are made in the direction of the venous blood and lymph stream. Again, effleurage can be thus performed, and very powerful strokes can be made, by the knuckles and the palmar aspect of the wrist. I place my knuckles upon this man's chest so, and I draw them towards myself softly, or with considerable force, just as I please, but the action is slow and deliberate. When I get to the end of my journey I raise my knuckles and depress my wrist, which I carry quickly thus over the ground that my knuckles have just traversed. In massaging you must remember that you can make the palmar aspect of your wrist the centre for some well-regulated and energising movements; for instance, if I wanted to massage deeply and with considerable pressure, I should place the palmar aspect of my wrist upon the part to be massaged, with my finger-tips horizontal to my wrist in this manner; I should then cause my wrist to partially rotate upon its own axis, guiding my fingers to perform a semicircle so. If you have a large and powerful thumb, you will find it exceedingly useful, and capable of good work; it is astonishing how much power can be exercised by the thumb. I place my finger-tips upon this man's chest, and I make them a fixed point; I then, you see, am able to work my thumb with considerable force and energy, in this manner, over a given area of about 90°. Well, now, there is another form of effleurage with which I am familiar, and which I find very useful for two reasons—first, because it readily covers a wide area, and secondly, because it affords a means of relief to the masseur. I have bared my arm purposely to show you exactly what I mean. Supposing I had been massaging this man's back by the process of effleurage and petrissage, but wished to continue with my work and my hands felt tired and cramped, I should then manipulate with the ulnar and flexor side of my arm in the manner I now show you. You will do well to bear this in mind, for I have frequently given myself great relief by adopting this mode of proceedure.

The next form of massage to which I must now draw your attention, is what we term petrissage. We look to this mode of manipulation to produce effects which no other form of massage can produce. I take this bit of skin between my thumb and forefinger, and I grip it firmly—in fact, I pinch it; I then roll it between my finger and thumb, in a manner somewhat similar to what one does in making a cigarette. This is the most simple demonstration of massage petrissage,—pinching, pressing, and squeezing are its essential and main attributes. If you want to petrissage a mass of muscle you grip it firmly in the palm of the hand, in the same way that you see me grip the muscle of this man's leg; you then make your way onwards or upwards from the periphery to the trunk (centripetally), like climbing up the rails of a ladder,—the same grip can, of course, be made thus between the fingers and the thumb, subjecting the parts to firm pressure, and rolling them and pinching them at the same time. By this you exert a powerfully exciting action upon the lymphatic system and the capillary system of vessels, and so render active a deep-seated and sluggish circulation, and stimulate the venous blood-flow onward towards the heart. When both hands are employed, they move simultaneously, and in the same direction, after this manner. As in all other forms of massage, the movements must be uniform, steady, and defined; don't jump about all over the limb, but proceed in the way you see me now—inch by inch, and without taking your hand from the part. By this means you cannot fail to rouse dormant nerve-energy, to make inactivity active, to produce a rise in temperature, and relieve dull and wearying muscular pains, and to restore nerve, energy, and power in the muscle This particular kind of massage relieves what is so common in deep-seated muscles, namely, muscular stagnation. By relieving this in the manner just described, you make the muscular structure receptive of the nerve-force which is sent to it, and thus it is rendered toned and active. This muscular stagnation may be instanced in a thousand ways, but it is particularly experienced by ladies at the change of life. When the circulation is interfered with muscular stagnation is one of the commonest symptoms; the arms feel heavy, so that to brush the hair can scarcely be accomplished; the legs feel so heavy, so wooden and lead-like, that it becomes a task to drag one leg after the other. Now this form of massage petrissage has been in my hands of signal service in these cases, and its use here is most simple; the muscles are relieved of congestion, and the normal nerve-force is expended equably and impartially to every demand made upon the nervous centres.

I cannot leave this form of massage, namely petrissage, without drawing your attention to some remarks upon this subject by Dr. Weir Mitchell, of Philadelphia, in a very interesting little book entitled *Fat and Blood, and how to make them.* On page 54 of this book (which I should advise you to get and read) you will find the following statement:—"After a few days of the milk diet with which my treatment ordinarily begins, the masseur or masseuse is set to work. An hour is chosen midway between two meals, and, the patient lying in bed, the manipulator starts at the feet." Here I will demonstrate to you upon this patient what Dr. Mitchell has put into words. I gently but firmly pinch up the skin of the foot, and roll it lightly between my fingers. I then, as you see, take each toe separately, and bend it at every joint. I then knead the muscles, and work with the finger-tips and the thumb between the bones, and I finish with the foot by seizing it with both hands and rolling it about so. Now I deal with the ankle, working away at all the crevices between the articulating bones, and putting the joint into every possible position. The leg is now treated in this way by surface pinching, then by deeper grasping of the areolar tissue, and last, by industrious and deeper pinching of the large muscular masses of the calf, and for this purpose I put the l'mb in a position of the utmost relaxation. You will observe that my grasp of the muscles is momentary, and that I use both my hands for the large muscles of the calf and thigh, the one contracting as the other loosens its grip. In treating the firm muscles in front of the leg, the fingers are made to roll the muscle under the cushions of the finger-tips. At brief intervals I seize the limb in both hands, and lightly run the grasp upwards, so as to favour the flow of venous blood-currents, and I then return again to the kneading of the muscles. I have now massaged this limb according to the directions laid down by Dr. Mitchell, and it is as perfect as any mode of massage can be; and you can judge for yourself if it is not the outcome of practical experience.

The last form of massage which I think it worth my while to bring before your notice is called *tapotement*, which means tapping, or percussing, or flagellating. The tapping may be done with the hand nearly closed, as mine is now, or it may be done with an indiarubber ball fixed to the end of a piece of cane, or it may be done with a cat-of-nine-tails, or by the aid of Dr. Granville's perceutor. You know what an astonishing effect tickling has upon a child. For instance, a child is moody, peevish, and fretful, due, probably, to the inhibition of some nerve by dyspepsia or constipated bowels. If you threaten the child with punishment, or send it to bed, or give it some task, you are really feeding the child's disease, whereas if, on the other hand, you cheer up the little thing, and if you give it a good tickling, you stimulate the child's nervous system; it actually laughs until it cries; its kidneys are stimulated, and its bowels too, and its bad temper vanishes.

The great value of tapotement is for the muscles of the loins and the back. You cannot well get at the deeper layers of these muscles by pinching or even kneading, but you can stimulate them by thumping and percussion in the manner I now show you. . . . After the parts have been massaged, I quite agree with Dr. Weir Mitchell that they should be lubricated with fresh cocoa oil, which keeps the skin in a smooth, soft, and supple state, and also helps the part to retain the heat which has been generated. I have before mentioned, as soon as a part has been manipulated it should be covered with flannel. There is also another point referred to by Dr. Weir Mitchell, which is borne out in my own practice, namely, that "the early use of massage is apt in some nervous women to cause increased nervousness, and even loss of sleep, but these symptoms may safely be disregarded, because they pass away in a few days, and very soon the patient begins to find the massage delightfully soothing, and to complain when it is omitted." I could say a great deal to you about the value of electricity in connection with massage, and my growing experience leads me to the conclusion that massage and electricity should always be employed in association. If I get hold of a difficult case which does not yield to electricity alone, I employ massage, and, by the aid of the two, I am usually successful, and, on the other hand, if massage alone does not answer, I associate it with electricity, and with like results. You will do well to get my colleague's (Dr. Herbert Tibbits) little book on *Electrical and Anatomical Demonstrations;* it is published by Churchill, of New Burlington Street, and is a perfect mine of wealth in all that relates to the application of electricity and galvanism to the human body; it will teach you in the most simple and concise manner possible when, where, and how to apply it.

